# Leptin/OB-R pathway promotes IL-4 secretion from B lymphocytes and induces salivary gland epithelial cell apoptosis in Sjögren's syndrome

**DOI:** 10.18632/oncotarget.18823

**Published:** 2017-06-28

**Authors:** Ting Xu, Wen Xie, Yingchun Ma, Shiliang Zhou, Lu Zhang, Jinyun Chen, Mingyuan Cai, Rurong Sun, Peirong Zhang, Shaobo Yu, Zheng Xu, Wanlan Jiang, Min Wu

**Affiliations:** ^1^ Department of Rheumatology and Immunology, The Third Affiliated Hospital of Soochow University, Jiangsu, Changzhou 213003, China

**Keywords:** Sjögren’s syndrome, SGECs, B lymphocytes, leptin, leptin receptor

## Abstract

Sjögren's syndrome (SjS) is a chronic autoimmune epithelitis in which cell apoptosis promotes the formation of inflammatory lesions. We used immunohistochemistry and TUNEL to assay B cell infiltration and apoptosis in salivary gland tissue from 16-week-old NOD/LtJ mice with SjS. In co-cultures of primary salivary glandepithelial cells (SGECs) and spleen B cells, we assessed SGEC viability and apoptosis using CCK8 assays and flow cytometry. ELISAs were employed to assess cytokine levels in culture medium. Leptin protein, leptin receptor (OB-R), pro- and anti-apoptotic proteins, and Jak2/Stat3/ERK signaling molecules were analyzed using western blotting. B cell infiltration and salivary gland apoptosis were increased in salivary tissue from mice with SjS. Leptin treatment had no effect on cell viability or apoptosis among B cells and primary SGECs. B cell and SGEC co-culture systems showed that leptin increased apoptosis induced by B lymphocytes, reduced SGEC cell viability, and promoted IL-4 secretion from B cells. This suggests Leptin/OB-R signaling stimulates B cells-induced SGEC apoptosis via IL-4 secretion and OB-R-Jak2-Stat3 activation.

## INTRODUCTION

Sjögren's syndrome (SjS) is a common autoimmune disorder characterized by the infiltration of lacrimal and salivary glands (SGs) by mononuclear cells with subsequent destruction of the parenchymal tissue [1–4]. Patient cell and animal model approaches of SjS suggest loss of salivary gland homeostasis as a trigger for the autoimmune response that promotes further damage to the gland [5–8].

Damage to exocrine cells accompanies lymphocyte infiltrates of ductal epithelial cells (ECs), which results in oral and ocular dryness [9–15]. However, these aggregates evolve from mild to focal, focal to diffuse, and vary in composition according to the severity of the disease. Inflammatory and apoptosis mediators have aberrant expression in salivary gland epithelial cells from SjS patients and murine models [16–22]. Apoptosis promotes the pathogenesis of inflammatory lesions [23–27]. During apoptotic cell death, nuclear antigens such as Ro/SSA and La/SSB ribonucleoproteins gain access to the immune system, leading to the induction of autoantibody responses characteristic of SjS [28–31].

Leptin is primarily secreted by adipocytes, and circulates in lean subjects. Insulin, overfeeding, endotoxins, glucocorticoids, and cytokines increase leptin expression; fasting, testosterone, thyroid hormone, and exposure to cold temperature decrease leptin expression [32–34]. The leptin receptor (OB-R) activates Janus-activated kinase (JAK), signal transducers and activators of transcription (STAT), insulin receptor substrate, and the mitogen-activated protein kinase (MAPK) pathways [35–38]. The best-characterized pathway in leptin signaling is the JAK/STAT pathway. The function of dysregulated leptin/OB-R signaling in SjS is not as well-understood.

Our previous study showed that leptin transcripts were elevated in B lymphocytes, while parenchymal cells contained more OB-R transcripts than lymphocytes or macrophages in SjS [39]. Here, we investigated the role of the leptin/OB-R signaling pathway in B lymphocyte-induced apoptosis of SGECs.

## RESULTS

### B lymphocytic infiltration and apoptosis in salivary gland tissue in mice with SjS

**Figure 1:**
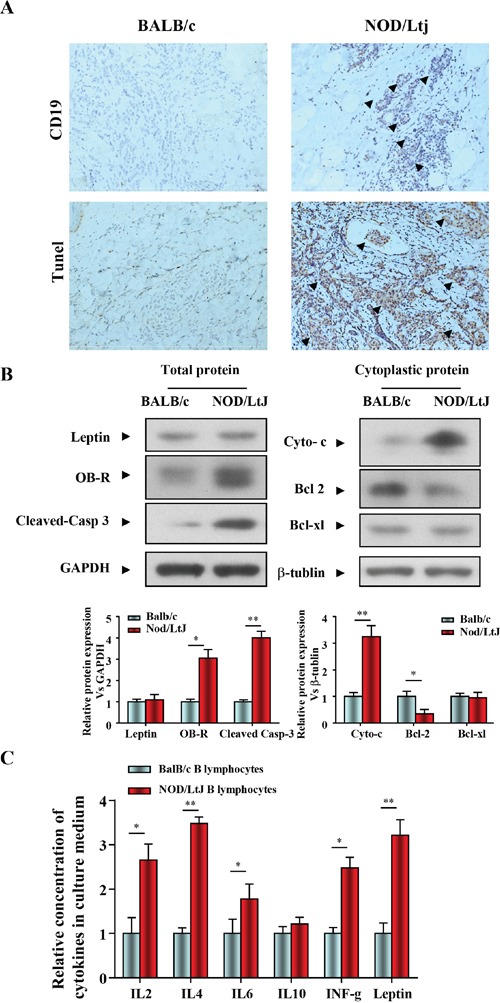
B lymphocytic infiltration and apoptosis in salivary gland tissue in mice with SjS **(A)** The levels of CD19 and apoptosis in salivary gland tissue were quantified by IHC and TUNEL (n=5, 16 wk). **(B)** Leptin, OB-R, pro- and anti-apoptotic proteins, cleaved caspase 3, cytochrome c, bcl2, and bcl-xl were analyzed using western blotting. Each sample was analyzed in duplicate, and expression was normalized to GAPDH. **(C)** Cytokine levels in culture medium were quantified by ELISA, and each sample was analyzed in duplicate. All values represent mean±SEM from 3 to 6 independent experiments. *P<0.05, **P<0.01.

In order to evaluate B lymphocytic infiltration and apoptosis in salivary gland tissue of diseased mice, a NOD/LtJ murine model of SjS was used. Immunohistochemical staining of salivary glands with specific anti-mouse CD19 antibodies showed a weak labeling signal in BalB/c mice, and a strong labeling signal in 16-week-old NOD/LtJ mice (Figure 1A). Salivary gland apoptosis was more extensive in NOD/LtJ mice than in BalB/c mice, and the TUNEL-positive area was adjacent to the lymphocytic infiltration (Figure 1A). These indicate the TUNEL assay results were consistent with B lymphocytic infiltration.

We then determined the role of the leptin/OB-R signaling pathway in salivary glands apoptosis in in mice with SjS. Leptin, OB-R, pro- and anti-apoptotic proteins, cleaved caspase 3, cytochrome c, bcl2 and bcl-xl were analyzed using western blotting. We found that both BALB/c and NOD/LtJ salivary gland tissues showed low leptin expression, while OB-R expression was increased. (Figure 1B). Consistent with the OB-R expression, the protein level of cleaved-caspase 3 and cytoplasmic cytochrome c were increased and bcl2 was decreased (Figure 1B). In contrast, the protein level of the anti-apoptotic protein bcl-xl was unchanged, which suggested that apoptosis was independent of bcl-xl.

Spleen B lymphocytes were isolated from BalB/c and NOD/LtJ mice, and the levels of cytokines released into the culture medium were measured using an ELISA multi-analyte assay (Figure 1C). The culture medium of NOD/LtJ B lymphocytes showed higher levels of inflammation-related cytokines and leptin than that of BalB/c B lymphocytes.

### Leptin treatment had no effect on cell viability and apoptosis in B lymphocytes and primary salivary epithelial cells

**Figure 2:**
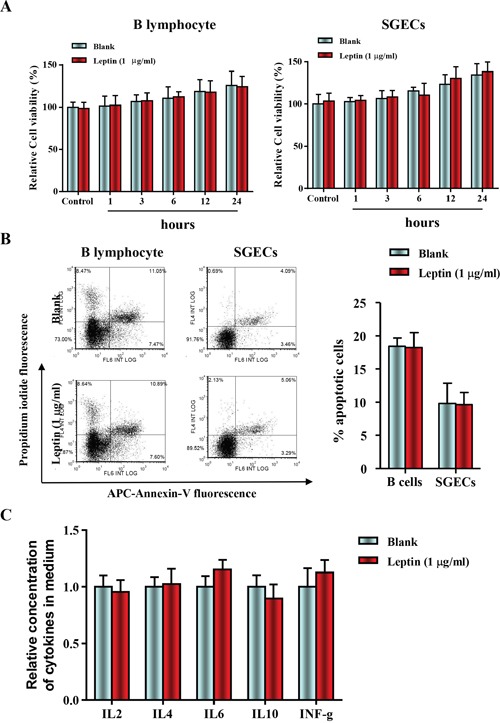
Leptin treatment had no effect on cell viability and apoptosis in B lymphocytes and primary salivary epithelial cells **(A)** The cell viability of SGECs was analyzed by the CCK8 assay. **(B)** The level of apoptosis in SGECs was analyzed by flow cytometry. **(C)** Cytokine levels in culture medium were quantified by ELISA, and each sample was analyzed in duplicate. All values represent mean±SEM from 3 to 6 independent experiments.

SGECs and B lymphocytes were isolated from 16-week-old NOD/LtJ mice, then cultured and incubated with or without leptin (1 mg/ml) for 1, 3, 6, 12, and 24 h. The leptin treatment did not affect cell viability, as shown by the CCK8 assay (Figure 2A). Apoptosis levels were also unaffected, as shown by AnnexinV/PI analysis (Figure 2B). Furthermore, levels of B lymphocyte-secreted cytokines, IL-2, IL-4, IL-6, IL-10, and IFN-γ were unchanged. These results suggest that the leptin/OB-R signaling pathway may not act directly on SGECs without B lymphocytes.

### Leptin treatment reduced SGEC viability, but increased B lymphocyte-induced apoptosis and the B lymphocyte secretion of IL-4

**Figure 3:**
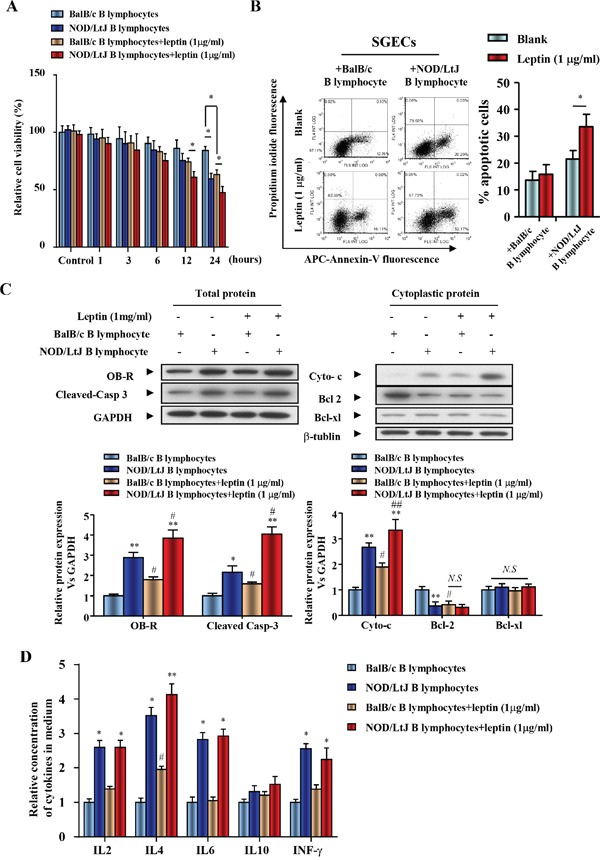
Leptin treatment reduced SGEC viability, increased apoptosis induced by B lymphocytes, and promoted the secretion of IL-4 from B lymphocytes **(A)** The viability of SGECs was analyzed by the CCK8 assay. **(B)** The level of apoptosis in SGECs was analyzed by flow cytometry. **(C)** OB-R, pro-apoptotic and anti-apoptotic proteins, cleaved caspase 3, cytochrome c, bcl2 and bcl-xl were analyzed using western blotting. **(D)** Cytokine levels in culture medium were quantified by ELISA. Each sample was analyzed in duplicate, and expression was normalized to GAPDH. *P<0.05, **P<0.01 vs. corresponding BalB/c B lymphocyte co-culture; ^#^*P*<0.05 vs. corresponding no leptin treatment group; *N.S*, not significant.

Due to the negative effect of leptin treatment on B lymphocytes and SGECs, a co-culture system was used to investigate the effect of leptin treatment on B lymphocyte-induced apoptosis. Because cell contact is necessary for B lymphocyte-induced SGECs apoptosis, SGECs and B lymphocytes were placed in the upper and lower chambers, respectively. Cell viability of SGECs co-cultured with B lymphocytes from NOD/LtJ mice decreased when compared with co-cultivation with B lymphocytes from BalB/c mice (Figure 3A).

A 24 h leptin (1 μg/ml) treatment decreased the cell viability of SGECs co-cultured with BalB/c B lymphocytes (Figure 3A). Leptin treatment also further reduced cell viability of NOD/LtJ B lymphocytes co-cultured with SGECs (Figure 3A). AnnexinV fluorescence showed that SGEC apoptosis increased in both BalB/c and NOD/LtJ B lymphocyte co-cultures (Figure 3B). Leptin, OB-R, pro- and anti-apoptotic proteins, cleaved caspase 3, cytochrome c, bcl2, and bcl-xl, were analyzed using western blotting. OB-R, cleaved-caspase 3, and cytoplasmic cytochrome c increased in SGECs after leptin treatment, while bcl2 decreased following treatment (Figure 3C). This indicates that the leptin/OB-R signaling pathway promotes B lymphocyte-induced SGECs apoptosis in SjS. In contrast, the protein level of the anti-apoptotic protein bcl-xl was unchanged, which suggests that bcl-xl has no effect on B lymphocyte-induced SGECs apoptosis in SjS.

To evaluate the levels of inflammatory cytokines produced from B lymphocytes after leptin treatment, IL-2, IL-4, IL-6, IL-10, and IFN-γ were analyzed by ELISA and flow cytometry (Figure 3D and Supplementary Figure 2). In both BalB/c and NOD/LtJ B lymphocytes, 24 h leptin treatment increased only IL-4 levels. These results suggest that the leptin/OB-R pathway stimulates B lymphocyte-induced SGECs apoptosis. In addition, leptin-induced IL-4 secretion from B lymphocytes may promote B lymphocyte-induced apoptosis.

### IL-4 increased OB-R expression and promoted the effect of leptin in enhancing SGECs apoptosis induced by B lymphocytes

**Figure 4:**
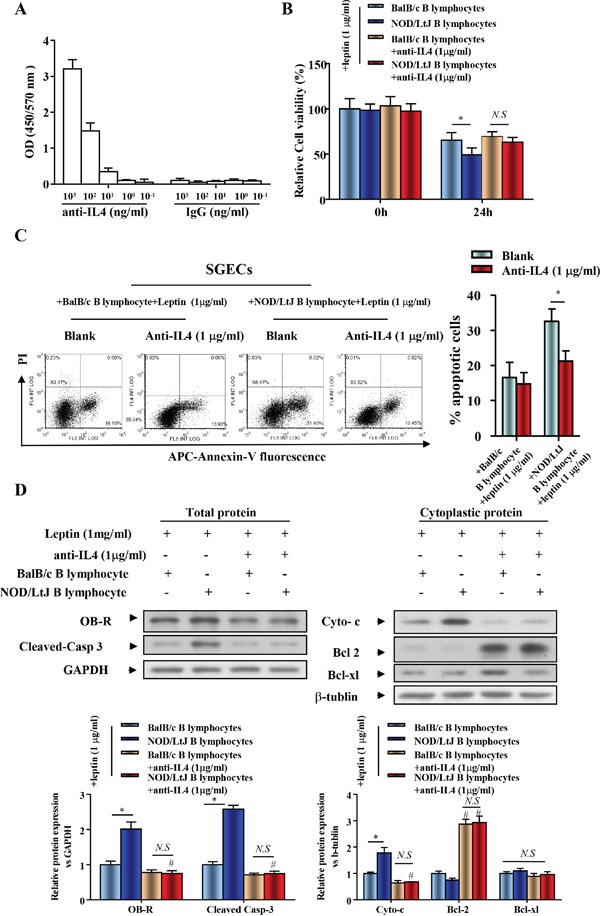
IL-4 increased expression of OB-R and ptomoted the effect of leptin on enhanced SGECs apoptosis induced by B lymphocytes **(A)** IL-4 ELISAs were performed with an anti-IL4 antibody. Data represent an average of duplicate measurements and SEM is shown. **(B)** The viability of SGECs was analyzed by the CCK8 assay. **(C)** The level of apoptosis in SGECs was analyzed by flow cytometry. **(D)** OB-R, pro-apoptotic and anti-apoptotic proteins, cleaved caspase 3, cytochrome c, bcl2, and bcl-xl were analyzed using western blotting. Each sample was analyzed in duplicate, and expression was normalized to GAPDH. *P<0.05, **P<0.01 vs. corresponding BalB/c B lymphocytes co-culture; ^#^*P*<0.05 vs. corresponding no leptin treatment group; *N.S*, not significant.

To investigate the role of IL-4 in the co-culture system, an anti-IL4 antibody was used to neutralize IL-4. To verify the binding of anti-IL4 to IL4, we performed an IL-4 ELISA. We found that anti-IL4 antibody bound to IL4 in a dose-dependent manner (Figure 4A). Following IL-4 neutralization, cell viability of leptin-treated SGECs, which were co-cultured with B lymphocytes from BalB/c or NOD/LtJ mice, was reversed to a similar level of cell viability as seen in leptin untreated and BalB/c B lymphocytes co-cultured with SGECs (Figure 4B). Following IL-4 neutralization, the initial increased apoptotic level of BalB/c B lymphocytes co-cultured SGECs was unchanged, but a complete reverse was observed in NOD/Ltj B lymphocytes co-cultured SGECs (Figure 4C).

We then verified that IL-4 neutralization reversed B lymphocyte-induced SGEC apoptosis using western blotting. The increased expression of OB-R in SGECs was completely reversed after IL-4 neutralization in the co-culture system (Figure 4D). This finding indicates the increased OB-R expression is correlated with the secretion of IL-4 from B lymphocytes. Cleaved-caspase 3 and cytoplasmic cytochrome c levels decreased after IL-4 neutralization, while bcl2 levels increased (Figure 4D).

### OB-R promotes B lymphocyte-induced apoptosis of SGECs via activating the Jak2-Stat3 signaling pathway in SjS

**Figure 5:**
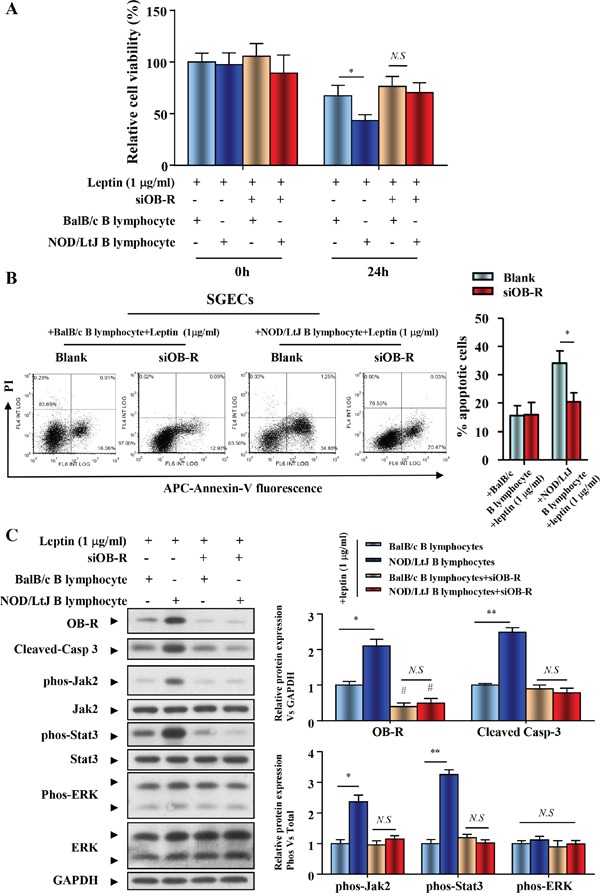
OB-R promotes B lymphocyte-induced apoptosis of SGECs in SjS **(A)** The cell viability of SGECs was analyzed by the CCK8 assay. **(B)** The level of apoptosis in SGECs was analyzed by flow cytometry. **(C)** OB-R, cleaved caspase 3, and activation of Jak2/Stat3 and ERK1/2 were analyzed using western blotting. Each sample was analyzed in duplicate, and expression was normalized to GAPDH. *P<0.05, **P<0.01 vs. corresponding BalB/c B lymphocytes co-culture; ^#^*P*<0.05 vs. corresponding no leptin treatment group; *N.S*, not significant.

To determine if increased OB-R expression is required for the leptin-induced increase in SGECs apoptosis, we generated a hairpin RNAi that selectively suppressed OB-R expression. SGECs transfected with OB-R siRNA showed decreased OB-R expression. Cell viability of leptin-treated SGECs, which were co-cultured with BalB/c or NOD/LtJ B lymphocytes, was reversed to similar levels seen in untreated BalB/c B lymphocytes co-cultured with SGECs (Figure 5A). Following OB-R down-regulation, the increased apoptosis was unchanged in co-cultured BalB/c B lymphocytes, and completely reversed in co-cultured NOD/Ltj B lymphocytes (Figure 5B).

Expression of both total and phosphorylated Jak2, Stat3, and ERK was investigated in cellular extracts from SGECs immediately after their co-cultivation with B lymphocytes. The Jak2-Stat3 activation comparablly increased, with the raised expression of OB-R and cleaved caspase 3 in the NOD/LtJ B lymphocyte co-culture group (Figure 5C). The down-regulation of OB-R with specific siRNAs reversed the expression levels of phosphorylated Jak2 and Stat3 to a similar level to that in the scramble control. These results suggest that OB-R expression promotes the activation of the Jak2-Stat3 signaling pathway (Figure 5C), as well as B lymphocyte-induced apoptosis of SGECs in SjS.

## DISCUSSION

We investigated B lymphocytic infiltration and apoptosis in salivary gland tissue in mice with SjS. Leptin secretion in B lymphocytes and OB-R expression were increased in NOD/LtJ SjS model mice. As apoptosis is thought to be an important mechanism in the pathogenesis of SjS, we then analyzed the effect of leptin treatment on B lymphocytes and primary SGECs. Leptin treatment had no effect on cell viability and apoptosis in B lymphocytes and primary SGECs.

Our previous study has found that the expression of the leptin receptor OB-R protein was specifically increased in parenchymal tissue during early and advanced stage disease of NOD/Ltj animals [39]. Moreover, the difference in cellular source of leptin and OB-R in SjS suggest that the leptin expression by B cells and OB-R expression by parenchymal tissue may play a crucial role in the initiation and development of SjS. As we failed to detect the cytokines seretion in SEGCs with or without leptin treatment (data not shown), in this study, we suppose and verify that leptin/OB-R signaling stimulates B cells-induced SGEC apoptosis via IL-4 secretion and Jak2-Stat3 activation. Briefly, to determine whether the leptin signaling pathway contributes to salivary gland apoptosis in SjS, a B lymphocyte and SGEC co-culture system was developed to evaluate the effect of leptin treatment on B lymphocyte-induced SGEC apoptosis. Initially, we found that leptin treatment enhanced the reduction in SGEC viability and apoptosis induced by B lymphocytes, and promoted the secretion of IL-4 from B lymphocytes. We evaluated the role of IL-4 by neutralizing IL-4 and found that IL-4 is a crucial cytokine, which mediatesthe increased expression of OB-R and contributes to the effect of leptin in enhanced SGECs apoptosis induced by B lymphocytes. Finally, by down-regulating OB-R it was found that OB-R is responsible for leptin enhanced and IL-4 mediated apoptosis in SjS.

Apoptotic death of ECs has been described by three different mechanisms [9–15]. First, ECs may be intrinsically activated and die due to membrane folding through autocrine Fas/FasL interactions. Second, ECs may directly interact with neighboring B or T lymphocytes and undergo apoptosis, again through Fas/FasL interactions. Third, activated cytotoxic B and T lymphocytes may release autoantibodies and cytokines, with ensuing activation of the caspase cascade [6, 8, 14–19, 22]. Our data reinforce the suggestion that B lymphocytes induce EC apoptosis; however, this is the first time that the role of a leptin/OB-R-dependent B lymphocyte apoptotic pathway has been established. This process requires secretion of IL-4 from B lymphocytes, which induces caspase 3 activation, promotes the phosphorylation of Jak2-Stat3, and results in apoptosis of SGECs.

B-lymphocyte derived cytokines, including IL-2, IL-4, IL6, IL10 and IFN-γ are likely to serve as effectors [16, 21, 22]. B lymphocytes act as antigen-presenting cells [40–42], behave as autophagic cells to bridge the gap between innate and adaptive responses, and regulate the formation of germinal centers via the production of lymphotoxins. Subsequently, these B lymphocytes have potential as therapeutic targets.

In the present study, we initially evaluated the role of Jak2-Stat3 signaling, but not ERK1/2 signaling, in the regulation of B lymphocytes-induced SGECs apoptosis.

**Figure 6:**
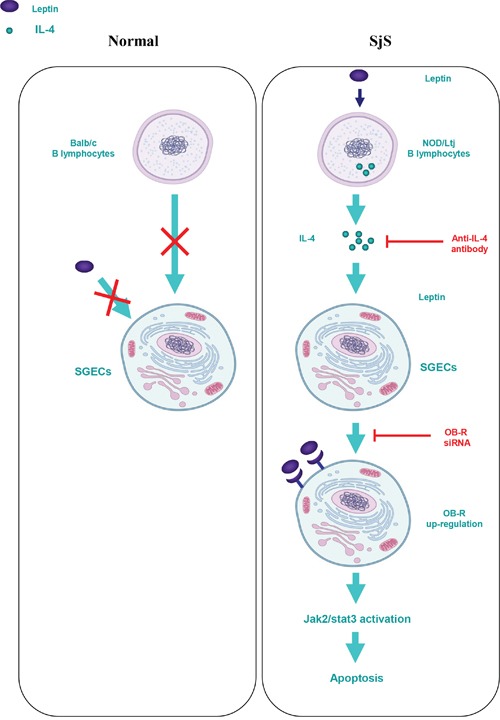
Schematic diagram for the proposed leptin/OB-R signaling exerts a pro-apoptotic effect on B lymphocyte-induced SGEC apoptosis via IL-4 secretion in SjS

We conclude that leptin/OB-R signaling exerts a pro-apoptotic effect on B lymphocyte-induced SGEC apoptosis in SjS. This effect is dependent on elevated IL-4 secretion from B lymphocytes, and the subsequent up-regulation of the OB-R-Jak2-Stat3 signaling pathway (Figure 6). Thus, it is suggested that B lymphocytes secreted IL-4 and OB-R in salivary gland may be valuable as a biomarker for initial diagnosis and assessment of disease progression and severity. Moreover, either IL-4 neutralization or OB-R down-regulation/inhibition may be new potential treatments of Sjogren.

## MATERIALS AND METHODS

### Mice

BALB/c and NOD/LtJ (NOD) mice (Shanghai Laboratory Animal Research Center, Shanghai, China) were used in this study. The mice were housed in micro-isolator cages, and all food, water, and bedding were autoclaved prior to use. All experiments were approved by the institutional animal care and use committee (IACUC) and hospital guidelines.

### Isolation of salivary tissue

Salivary tissue was removed after euthanasia. The tissue was fixed in 10% buffered formalin or frozen for immunohistochemistry (IHC) analysis.

### Immunohistochemical analysis

The *in-situ* expression of CD19 in paraffin embedded labial minor salivary gland biopsy specimens was analyzed using the EnVision system (Dako, Glostrup, Denmark) and anti-CD19 monoclonal antibody (Abcam) at 1:200 dilution. Antigen retrieval was performed by microwaving in 10 mM citrate buffer (pH 6.0). Normal non-immune fetal bovine serum and 0.5% H_2_O_2_ in methanol were used to block nonspecific antibody binding and endogenous peroxidase activity, respectively. Negative control staining was performed by replacing the primary antibody with an irrelevant antibody.

### Quantification of cytokines in the culture supernatants by ELISA

The concentrations of IL-2, IL-4, IL-6, IL-10, INF-γ, and leptin secreted into serum or culture supernatants were quantified using ELISA test kits (R&D Systems, Minneapolis, MN, USA), according to the manufacturers’ instructions.

### TdT-mediated dUTP-biotin nick end-labeling (TUNEL) assay

The TUNEL method was used to detect apoptotic cells with the *in situ* cell apoptosis detection kit (Roche, Palo Alto, CA, USA), according to the manufacturer's instructions. Tissue sections from the phosphate buffered saline (PBS)-treated group were stained and served as negative controls.

### Isolation, culture and characterization of B lymphocytes

Spleens were minced and then filtered to remove fragments and clumps. Spleen samples were layered onto Ficoll-Hypaque and centrifuged. Mononuclear cells were incubated with neuraminidase-treated sheep red blood cells and T cells depleted by a second round of centrifugation. B cells were cultured in 96-well culture plates in RPMI-1640 medium (Invitrogen Life Technologies) supplemented with 10% heat-inactivated fetal calf serum, 2 mM L-glutamine, 200 U/ml penicillin, and 100 mg/ml streptomycin at 2×10^5^ cells/well.

Flow cytometry analyses were performed with Navios flow cytometer (Beckman Coulter, Miami, FL, USA). Purity of primary B lymphocytes was assessed by staining with anti-mouse IgG FITC (BD Biosciences, Palo Alto, CA, USA) or anti-mouse CD19 FITC (BD Biosciences). All preparations were >98% pure B lymphocytes (Supplementary Figure 1). To evaluate intracellular expression of cytokines (IL-2, IL-4, IL-6, IL-10, and IFN-γ), primary B lymphocytes was assessed by staining with anti-mouse IL-2 FITC, anti-mouse IL-4 FITC, anti-mouse IL-6 FITC, anti-mouse IL-10 FITC or anti-mouse IFN-γ FITC antibody (Abcam).

### Culture of primary salivary gland epithelial cells

The method used to culture SGECs from NOD/LtJ mice has been previously described (40). Briefly, tissues were rinsed with cold, sterile PBS containing 100 units/ml penicillin and 100 μg/ml streptomycin. Then they were minced into fragments of about 1 mm^3^. One fragment of each tissue sample was placed in a 24-well plate coated with type I collagen (Iwaki, Tokyo, Japan), and cultured in defined keratinocyte–SFM with growth supplement, 0.4 μg/ml hydrocortisone, 100 units/ml penicillin, 100 μg/ml streptomycin, and 25 μg/ml bovine pituitary extract. Epithelial cell outgrowth from the explant was observed after 1–2 weeks. After reaching confluence, the monolayer cells were trypsinized and subcultured. Cultured epithelial cells were used for the experiments when they reached 90% confluence. All experiments used passage 3 cells.

### Co-culture of B-lymphocytes with SGECs

SGECs were seeded into 96-well flat-bottomed culture plates. When the cells reached confluence, 1.5×10^5^ B lymphocytes were added to 1 ml RPMI-1640 medium (Lonza) supplemented with 10% FCS, 2 mM L-glutamine, and antibiotics. To avoid allogenic response and long-term culture artifacts, the cells were collected 24 h later. Transwells (Corning, NY, USA) enabled verification of whether cell–cell contacts were discernable. To address this issue, confluent SGECs were placed in the lower chamber and B-lymphocytes in the upper chamber.

### Cell viability assay and cell apoptosis detection

The cell viability of SGECs was determined using a CCK8 cell counting kit, and cell apoptosis was determined by flow cytometry. Briefly, SGECs were cultured in collagen type I-coated plates at 37°C for the indicated times. Ethanol-fixed cell suspensions were centrifuged, followed by the addition of 50 ml RNase solution and 450 ml propidium iodide (final concentration 20 mg/ml). The cells were washed twice and subsequently analyzed by flow cytometry (BD Biosciences, Palo Alto, CA, USA).

### Antibody specificity assay

Plates were pre-coated with recombinant mouse IL-4 (R&D Systems, Minneapolis, MN, USA). Primary antibodies were used as follows: mouse IgG isotype control and anti-IL-4 (Abcam). HRP-conjugated secondary antibody (goat anti-mouse, Jackson ImmunoResearch, West Grove, PA, USA) was added, and samples were developed with tetramethylbenzidine (TMB) substrate (BD Biosciences). Absorbance was read at 450/570 nm.

### Western blot analysis

Protein immune blotting was performed as described previously (39). Salivary gland tissues were collected and homogenized. Tissue lysate was separated by SDS-PAGE electrophoresis and then electrotransferred to polyvinylidene fluoride membranes (Millipore). The expression of leptin, OB-R, cleaved-caspase 3, cytochrome c, bcl2, and bcl-xl were analyzed using specific antibodies (Cell Signaling Technology, Beverly, MA, USA). After washing with PBST, the membranes were incubated with HRP-conjugated goat anti-rabbit IgG at room temperature for 1 h followed by washing with PBST. The target proteins were examined with an ECL system (Millipore), and visualized with autoradiography film. The ratios of gray values were analyzed by Quantity One software (Bio-Rad, Hercules, CA, USA).

### Statistical analysis

All experiments were performed in triplicate. The differences between the groups were determined by ANOVA analysis, and two groups were compared by the t-test using GraphPad Prism 5.0 (GraphPad Software, Inc., San Diego, CA, USA). A value of p < 0.05 was considered statistically significant.

## SUPPLEMENTARY FIGURES


