# Supplementary material for: Leptin/OB-R pathway promotes IL-4 secretion from B lymphocytes and induces salivary gland epithelial cell apoptosis in Sjögren's syndrome
Source: Oncotarget. 2017 Jun 28;8(38):63417–29. doi: 10.18632/oncotarget.18823 (PMC5609933; doi:10.18632/oncotarget.18823)
Supplement: Supplementary file 1 [file oncotarget-08-63417-s001.pdf]

Leptin/OB-R pathway promotes IL-4 secretion from B lymphocytes and induces salivary gland epithelial cell apoptosis in Sjögren's syndrome

SUPPLEMENTARY MATERIALS

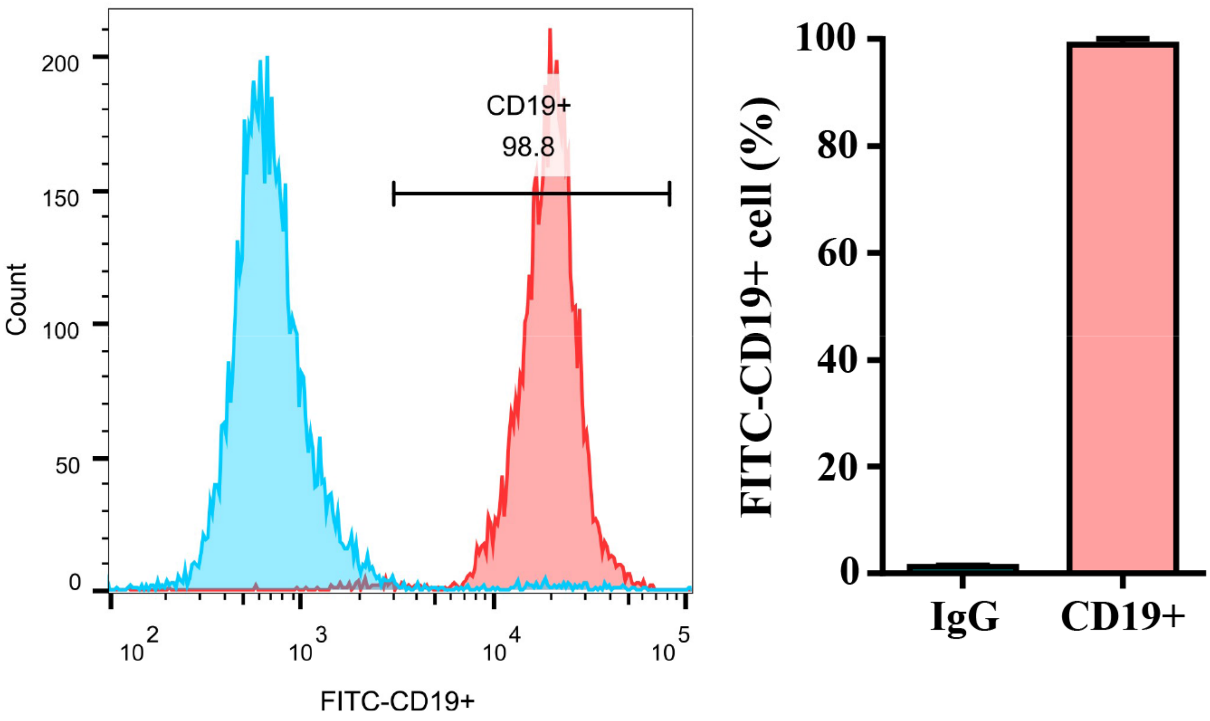

Supplementary Figure 1: Flow cytometric analysis of primary B lymphocytes stained with anti-CD19 PE antibody to assess purity.

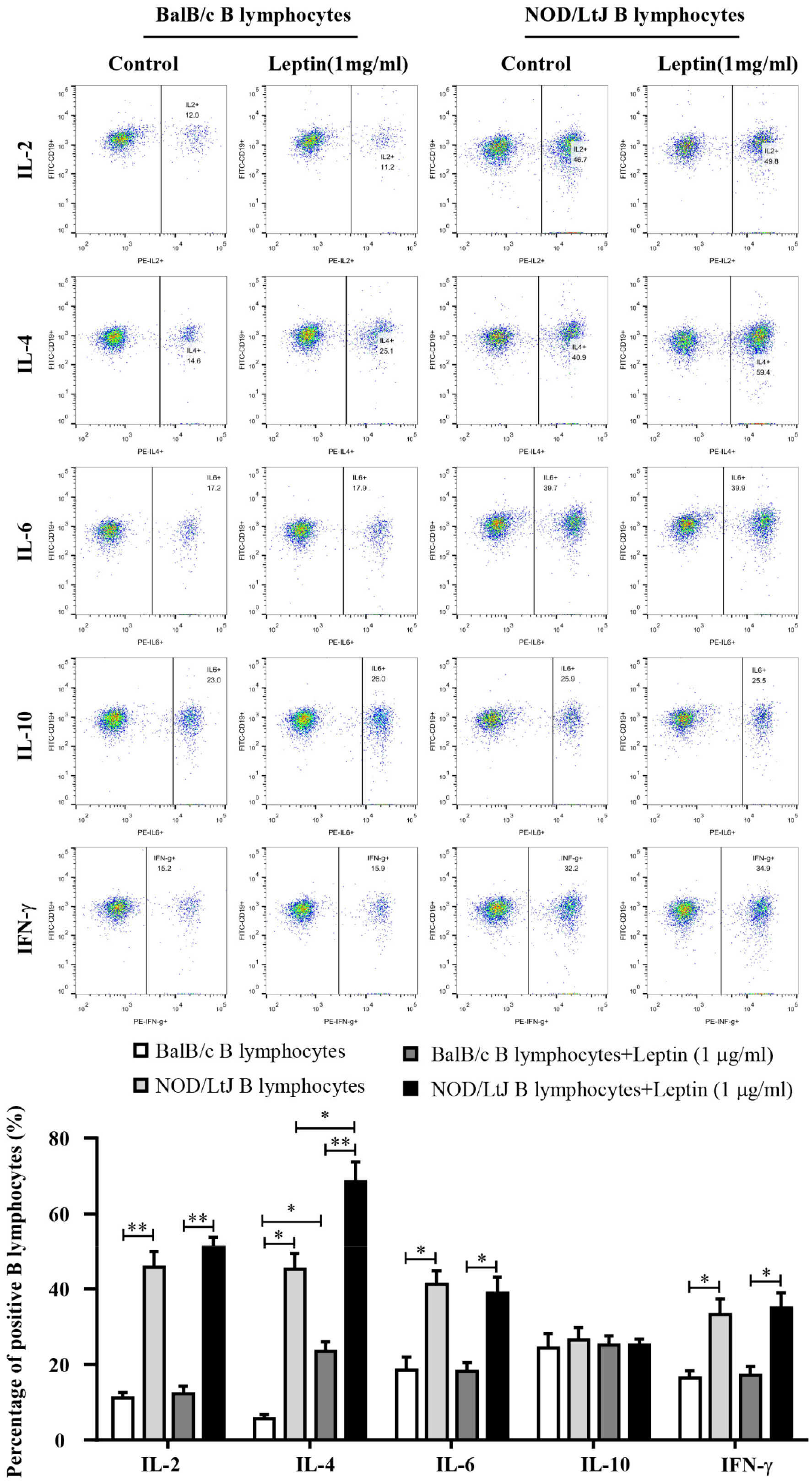

Supplementary Figure 2: Flow cytometric analysis of primary B lymphocytes stained with specific antibodies to assess the intracellular expression of cytokines (IL-2, IL-4, IL-6, IL-10, and IFN- γ).
